# Skin Cancer Classification Using Convolutional Neural Networks: Systematic Review

**DOI:** 10.2196/11936

**Published:** 2018-10-17

**Authors:** Titus Josef Brinker, Achim Hekler, Jochen Sven Utikal, Niels Grabe, Dirk Schadendorf, Joachim Klode, Carola Berking, Theresa Steeb, Alexander H Enk, Christof von Kalle

**Affiliations:** 1 National Center for Tumor Diseases Department of Translational Oncology German Cancer Research Center Heidelberg Germany; 2 Department of Dermatology University Hospital Heidelberg University of Heidelberg Heidelberg Germany; 3 Skin Cancer Unit German Cancer Research Center Heidelberg Germany; 4 Department of Dermatology, Venereology and Allergology University Medical Center Mannheim Ruprecht-Karl University of Heidelberg Heidelberg Germany; 5 Bioquant Hamamatsu Tissue Imaging and Analysis Center University of Heidelberg Heidelberg Germany; 6 Department of Dermatology University Hospital of Essen University of Duisburg-Essen Essen Germany; 7 Department of Dermatology University Hospital Munich Ludwig Maximilian University of Munich Munich Germany

**Keywords:** skin cancer, convolutional neural networks, lesion classification, deep learning, melanoma classification, carcinoma classification

## Abstract

**Background:**

State-of-the-art classifiers based on convolutional neural networks (CNNs) were shown to classify images of skin cancer on par with dermatologists and could enable lifesaving and fast diagnoses, even outside the hospital via installation of apps on mobile devices. To our knowledge, at present there is no review of the current work in this research area.

**Objective:**

This study presents the first systematic review of the state-of-the-art research on classifying skin lesions with CNNs. We limit our review to skin lesion classifiers. In particular, methods that apply a CNN only for segmentation or for the classification of dermoscopic patterns are not considered here. Furthermore, this study discusses why the comparability of the presented procedures is very difficult and which challenges must be addressed in the future.

**Methods:**

We searched the Google Scholar, PubMed, Medline, ScienceDirect, and Web of Science databases for systematic reviews and original research articles published in English. Only papers that reported sufficient scientific proceedings are included in this review.

**Results:**

We found 13 papers that classified skin lesions using CNNs. In principle, classification methods can be differentiated according to three principles. Approaches that use a CNN already trained by means of another large dataset and then optimize its parameters to the classification of skin lesions are the most common ones used and they display the best performance with the currently available limited datasets.

**Conclusions:**

CNNs display a high performance as state-of-the-art skin lesion classifiers. Unfortunately, it is difficult to compare different classification methods because some approaches use nonpublic datasets for training and/or testing, thereby making reproducibility difficult. Future publications should use publicly available benchmarks and fully disclose methods used for training to allow comparability.

## Introduction

In the past 10-year period, from 2008 to 2018, the annual number of melanoma cases has increased by 53%, partly due to increased UV exposure [1,2]. Although melanoma is one of the most lethal types of skin cancer, a fast diagnosis can lead to a very high chance of survival.

The first step in the diagnosis of a malignant lesion by a dermatologist is visual examination of the suspicious skin area. A correct diagnosis is important because of the similarities of some lesion types; moreover, the diagnostic accuracy correlates strongly with the professional experience of the physician [3]. Without additional technical support, dermatologists have a 65%-80% accuracy rate in melanoma diagnosis [4]. In suspicious cases, the visual inspection is supplemented with dermatoscopic images taken with a special high-resolution and magnifying camera. During the recording, the lighting is controlled and a filter is used to reduce reflections on the skin, thereby making deeper skin layers visible. With this technical support, the accuracy of skin lesion diagnosis can be increased by a further 49% [5]. The combination of visual inspection and dermatoscopic images ultimately results in an absolute melanoma detection accuracy of 75%-84% by dermatologists [6,7].

For some time, the problem of classifying skin lesions has also moved into the focus of the machine learning community. Automated lesion classification can both support physicians in their daily clinical routine and enable fast and cheap access to lifesaving diagnoses, even outside the hospital, through installation of apps on mobile devices [8,9]. Before 2016, research mostly followed the classical workflow of machine learning: preprocessing, segmentation, feature extraction, and classification [9-11]. However, a high level of application-specific expertise is required, particularly for feature extraction, and the selection of adequate features is very time-consuming. In addition, errors and the loss of information in the first processing steps have a very strong influence on the classification quality. For example, a poor segmentation result often leads to poor results in feature extraction and, consequently, low classification accuracy.

In 2016, a change occurred regarding the research of lesion classification techniques. An indication of this change can be found in the methods submitted to the 2016 International Symposium on Biomedical Imaging (ISBI) [12]. The 25 participating teams did not apply traditional standard machine learning methods; instead, they all employed a deep learning technique: convolutional neural networks (CNNs) [13].

This paper presents the first systematic review of the state-of-the-art research on classifying skin lesions using CNNs. The presented methods are categorized by whether a CNN is used exclusively as a feature extractor or whether it is applied for end-to-end-learning. The conclusion of this paper discusses why the comparability of the presented techniques is very difficult and which challenges must be addressed in the future.

## Methods

### Search Strategy

The Google Scholar, PubMed, Medline, ScienceDirect, and Web of Science databases were searched for systematic reviews and original research articles published in English. The search terms *convolutional neural networks*, *deep learning*, *skin cancer*, *lesions*, *melanoma*, and *carcinoma* were combined. Only papers that showed sufficient scientific proceedings are included in this review.

### Study Selection

We limited our review to skin lesion classification methods. In particular, methods that apply a CNN only for lesion segmentation or for the classification of dermatoscopic patterns as in Demyanov et al [14] are not considered in this paper. Furthermore, only papers that show a sufficient scientific proceeding are included in this review. This latter criterion includes presenting the approaches in an understandable manner and discussing the results sufficiently. Works in which the origin of the performance was not plausible are not considered in this work, for example, in Carcagnì et al [15] or Dorj et al [16].

### Convolutional Neural Networks

CNNs are neural networks with a specific architecture that have been shown to be very powerful in areas such as image recognition and classification [17]. CNNs have been demonstrated to identify faces, objects, and traffic signs better than humans and therefore can be found in robots and self-driving cars.

CNNs are a supervised learning method and are therefore trained using data labeled with the respective classes. Essentially, CNNs learn the relationship between the input objects and the class labels and comprise two components: the hidden layers in which the features are extracted and, at the end of the processing, the fully connected layers that are used for the actual classification task. Unlike regular neural networks, the hidden layers of a CNN have a specific architecture. In regular neural networks, each layer is formed by a set of neurons and one neuron of a layer is connected to each neuron of the preceding layer. The architecture of hidden layers in a CNN is slightly different. The neurons in a layer are not connected to all neurons of the preceding layer; rather, they are connected to only a small number of neurons. This restriction to local connections and additional pooling layers summarizing local neuron outputs into one value results in translation-invariant features. This results in a simpler training procedure and a lower model complexity.

### Current Classifiers for Skin Lesions Based on Convolutional Neural Networks

**Figure 1:**
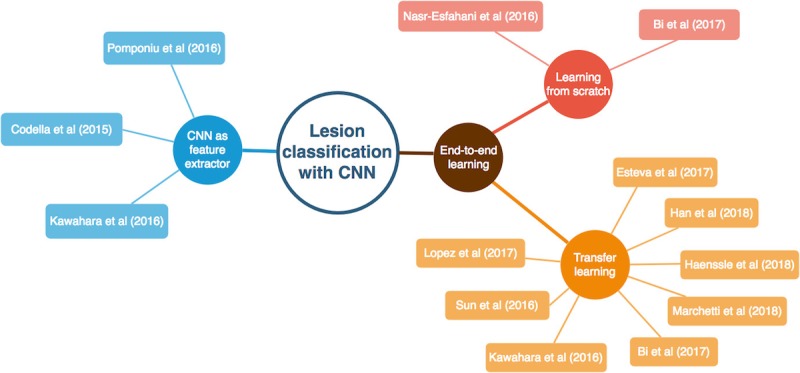
An overview of the presented convolutional neural networks (CNNs) methods and the corresponding categorization.

In this section, the individual CNN methods used to classify skin lesions are presented. CNNs can be used to classify skin lesions in two fundamentally different ways. On the one hand, a CNN pretrained on another large dataset, such as ImageNet [18], can be applied as a feature extractor. In this case, classification is performed by another classifier, such as k-nearest neighbors, support vector machines, or artificial neural networks. On the other hand, a CNN can directly learn the relationship between the raw pixel data and the class labels through end-to-end learning. In contrast with the classical workflow typically applied in machine learning, feature extraction becomes an integral part of classification and is no longer considered as a separate, independent processing step. If the CNN is trained by end-to-end learning, the research can be additionally divided into two different approaches: learning the model from scratch or transfer learning. An overview of the presented CNN methods is shown in Figure 1.

A basic requirement for the successful training of deep CNN models is that sufficient training data labeled with the classes are available. Otherwise, there is a risk of overfitting the neural network and, as a consequence, an inadequate generalization property of the network for unknown input data. There is a very limited amount of data publicly available for the classification of skin lesions. Almost all published methods use datasets that contain far less than 1000 training data points per training class. In comparison, well-known CNN models for image classification, such as AlexNet [18], VGG [19], GoogLeNet [20], or ResNet [21], are trained via the large image database ImageNet and have over 1000 training images for each training class.

However, through the use of a specific training procedure called transfer learning, powerful CNN models with several million free parameters can also be employed for classification, even if only a small amount of data are available for training. In this case, the CNN is pretrained using a very large dataset, such as ImageNet; it is then used as an initialization of the CNN for the respective task. In particular, the last fully connected layer of the pretrained CNN model is modified according to the number of training classes in the actual classification task. There are then two options for the weights of the pretrained CNN: to fine-tune all layers of the CNN or to freeze some of the front layers because of overfitting problems and to fine-tune only some back layers of the network. The idea behind this technique is that that the front layers of a CNN contain more generic features (eg, edge or color-blob detectors) that are useful for many tasks, but the back layers of the CNN become increasingly specific to the details of the classes contained in the original dataset.

In the following discussion, statistical quantities to evaluate different classifiers are introduced. Next, methods that utilize the CNN as a feature extractor are presented. The last subsection provides an overview of the methods involved when using CNN for end-to-end-learning.

### Performance Metrics for Classifiers

A classifier assigns each object to a class. This assignment is generally not perfect and objects may be assigned to the wrong class. To evaluate a classifier, the actual class of the objects must be known. To evaluate the classification quality, the class assigned by the classifier is compared with the actual class. This allows the objects to be divided into the following four subsets:

1. True positive (TP): the classifier correctly predicts the positive class.
2. True negative (TN): the classifier correctly predicts the negative class.
3. False positive (FP): the classifier incorrectly predicts the positive class.
4. False negative (FN): the classifier incorrectly predicts the negative class.

Based on the cardinality of these subsets, statistical quantities for the classifier can now be calculated. A common and widely used quantity is accuracy, which is only a reasonable measure if the different classes in the dataset are approximately equally distributed. Accuracy is calculated by (TP + TN)/(TP + TN + FP + FN). It specifies the percentage of objects that have been correctly classified.

Two other important metrics are sensitivity and specificity, which can be applied even if the different classes are not equally distributed. Sensitivity indicates the ratio of objects correctly classified as positive out of the total number of positive objects contained in the dataset and is calculated by TP/(TP + FN).

Specificity indicates the ratio of negative objects correctly classified as negative out of the total number of negative objects contained in the available dataset and is calculated by TN/(TN + FP).

The output of a binary classifier is interpreted as a probability distribution over the classes. Normally, objects with an output value greater than .5 are assigned to the positive class in a binary classifier and objects with an output value less than .5 are assigned to the negative class. An alternative approach is used based on the receiver operating characteristic (ROC). The threshold used for classification systematically varies between 0 and 1, and the sensitivity and specificity are determined for each selected threshold. The ROC curve is calculated by plotting the sensitivity against 1-specificity and can be used to evaluate the classifier. The further the ROC curve deviates from the diagonal, the better the classifier. A suitable overall measure for the curve is the area under the curve (AUC).

## Results

### Classifier That Utilizes the Convolutional Neural Network as a Feature Extractor

A CNN can be included in classification by removing the fully connected layers of a CNN that were pretrained with a large dataset. In skin lesion classification, pretraining is performed using ImageNet. Despite the nonmedical image domain, the learned features have sufficient quality for lesion classification [22].

Pomponiu et al [23] used only 399 images from a standard camera for the classification of melanomas versus benign nevi. First, data augmentation and preprocessing were performed. Subsequently, a pretrained AlexNet was applied for the extraction of representational features. The lesions were then classified with a k-nearest-neighbor classifier using cosine distance metrics. The algorithm was not tested with an independent test dataset; only a cross-validation was performed. The algorithm achieved a sensitivity of 92.1%, a specificity of 95.18%, and an accuracy of 93.64%. In addition to the nonexistent independent test dataset, it is also critical to note that the region of interest for each skin lesion must be manually annotated.

An AlexNet model for feature extraction was also applied by Codella et al [24]. In contrast to Gutman et al [12], however, a total of 2624 dermatoscopic images from the publicly available International Skin Imaging Collaboration (ISIC) database were used for the classification of melanoma versus nonmelanoma lesions or melanoma versus atypical nevi. In addition to the modified AlexNet outputs, the authors also used low-level handcrafted features and features from sparse coding, a deep residual network, and a convolutional U-network. Classification based on all of these features was then performed using a support vector machine. The authors reported an accuracy of 93.1%, a sensitivity of 94.9%, and a specificity of 92.8% for classifying melanoma versus nonmelanoma. In the more difficult discrimination between melanomas and atypical nevi, an accuracy of 73.9%, a sensitivity of 73.8%, and a specificity of 74.3% were reported. The authors also showed that the use of deep features results in a better performance compared to classifiers that only used low-level handcrafted features.

Kawahara et al [25] used a linear classifier to classify 10 different skin lesions. Feature extraction was also performed using an AlexNet whose last fully connected layer was replaced with a convolutional layer. This slightly modified AlexNet was tested using the public Dermofit Image Library, which contains 1300 clinical images of 10 skin lesions. An accuracy of 81.8% was achieved based on the entire dataset of 10 different types of skin lesions.

### Skin Lesion Classifier Using End-to-End Learning

#### Convolutional Neural Network Model Training With Transfer Learning

Because publicly available datasets are limited, a common method of skin lesion classification involves transfer learning. Therefore, all such works pretrain a CNN via the ImageNet dataset; next, the weighting parameters of the CNN are fine-tuned to the actual classification problem.

Esteva et al [26] presented a landmark publication. For the first time, a CNN model was trained with a large amount of data, specifically 129,450 images, of which 3374 were obtained from dermatoscopic devices and represented 2032 different skin lesions. Two binary classification problems were considered: keratinocyte carcinomas versus benign seborrheic keratosis and malignant melanomas versus benign nevi. The last classification differentiation was performed for both clinical and dermatoscopic images. The authors used a GoogLeNet Inception v3 model for the classification, which was pretrained with the large image database ImageNet. The CNN model was then fine-tuned to classify skin lesions using transfer learning. A special property of this approach is the use of a novel tree-structured disease taxonomy in which the individual diseases form the leaves of the tree. The inner nodes group together individual diseases that are visually and clinically similar. The CNN does not have a two-dimensional vector as the output; instead, it reports a probability distribution with over 757 training classes. To determine the probabilities of a coarser lesion class (ie, an inner node at a higher level in the tree), the probabilities of the child nodes of this coarser lesion class are summed together. The authors show within the evaluation that a CNN that has been trained for finer classes has a better performance than a CNN that has been trained for the distinct classes that are of interest for the problem. The trained CNN was tested with test data that were fully biopsy-proofed and achieved an ROC AUC of .96 for carcinomas, an ROC AUC of .96 for melanomas, and an ROC AUC of .94 for melanomas classified exclusively with dermatoscopic images.

Haenssle et al [3] presented a very similar approach to Esteva et al [26]. A GoogLeNet Inception v3 model was adapted for skin lesion classification with transfer learning, whereby the weights were fine-tuned in all layers. The analysis was limited to dermatoscopic images of melanoma versus benign nevi and the AUC ROC achieved for this task was .86 (Esteva et al [26]: .94). The exact number of training data points was not provided and not all data had been proofed by a biopsy. However, the publication included the largest number of dermatologists to date (n=58) and was the first to indicate that additional clinical information improves both; sensitivity and specificity of dermatologists.

Han et al [27] are particularly noteworthy for their scientific transparency since they have made their computer algorithm publicly available for external testing. The team presented a classifier for 12 different skin diseases based on clinical images. They developed a ResNet model that was fine-tuned with 19,398 training images. With the publicly available Asan dataset, the CNN model achieved ROC AUCs for the diagnoses of basal cell carcinoma, squamous cell carcinoma, intraepithelial carcinoma, and melanoma of .96, .83, .82, and .96, respectively.

An ensemble of CNNs for the classification of melanomas versus nevi or lentigines is presented by Marchetti et al [13]. They implemented five methods to fuse all automated predictions from the 25 participating teams in the ISBI 2016 Challenge into a single classification result. For this purpose, they tested two nonlearning approaches and three machine learning methods. The fusion algorithms were trained with 279 dermatoscopic images from the ISBI 2016 Challenge dataset and were tested with 100 other dermatoscopic images from the same dataset. Based on average precision, greedy fusion was the best-performing ensemble method with a sensitivity of 58% and a specificity of 88%.

Another type of CNN ensemble was presented by Bi et al [28]. They considered the classification of melanomas versus seborrheic keratosis versus nevi using dermatoscopic images. They did not train multiple CNNs for the same classification problem; instead, three ResNets for different problems were trained: one for the original three-class problem and two binary classifiers (ie, melanoma versus both other lesion classes or seborrheic carcinoma versus both other lesion classes) by fine-tuning a pretrained CNN. The test utilized 150 dermatoscopic images and resulted in an ROC AUC of .854 for melanomas, an ROC AUC of .976 for seborrheic carcinomas, and an average ROC AUC over all classes of .915.

A special architecture of a CNN ensemble is presented by Kawahara et al [29]. The CNN was composed of multiple parts in which each part considers the same image at a different resolution. Next, an end layer combines the outputs from multiple resolutions into a single layer. The CNN identifies interactions across different image resolutions and the weighting parameters are completely optimized by end-to-end learning. The algorithm achieved an average classification accuracy of 79.5% in the public Dermofit Image Library.

Similar to Esteva et al [26], Sun et al [30] introduced a classifier that used 198 very finely defined training classes. In total, 6584 clinical images of the publicly available image archive DermQuest were used for training and testing and the performance of the CNN models CaffeNet and VGGNet were evaluated for this classification problem. The best average accuracy of 50.27% over all 198 classes was obtained using a pretrained VGGNet, which was optimized by fine-tuning the weighting parameters.

A modified VGGNet was also utilized by Lopez et al [31], where the classification of melanoma versus nevi or lentigines was addressed using dermatoscopic images. The authors compared the classification accuracy of a CNN trained from scratch, a pretrained CNN with transfer learning and frozen layers, and a pretrained CNN with transfer learning and fine-tuning of the weighting parameters. All three configurations were tested with 379 images from the ISBI 2016 Challenge dataset, and the last-mentioned configuration achieved the highest accuracy of 81.33%.

#### Convolutional Neural Network Model Training From Scratch

The previously introduced two-step approach by Bi et al [28] also falls under the category “learning from scratch” due to the method of training of the the ResNet model for the three-class classification of melanoma versus seborrheic keratosis versus nevus. Bi et al [28] used approximately 3600 dermatoscopic images from the ISBI 2017 Challenge dataset and additional images from the ISIC Archive to achieve the results reported.

In Nasr-Esfahani et al [32], a two-layer CNN was trained from scratch for the distinction of melanoma versus benign nevi based on clinical images. Only 136 images were used to train the model and the test dataset contained only 34 images. The images were all from the public image archive of the Department of Dermatology of the University Medical Center Groningen. The method achieved a sensitivity of 81%, a specificity of 80%, and an accuracy of 81%. However, the result should be viewed critically because the test dataset was very limited.

## Discussion

### Principal Findings

One issue with the comparison of skin lesion classification methods is that the considered problem formulations of the individual works differ, sometimes only slightly. This occurs not only for the considered training classes and the used data, but also for the presented statistical quantities. In addition, some works use nonpublic archives of skin clinics in addition to publicly accessible data archives [3,26]. This makes it even more difficult to reproduce the results. Since 2016, the ISIC Melanoma Project has attempted to improve this aspect by establishing a publicly accessible archive of dermatoscopic skin lesion images as a benchmark for education and research [12]. In addition, they announced an annual challenge in which a clearly defined problem must be addressed. It would be desirable if more work would compare itself with this benchmark to achieve a better ranking of the procedures in the state of research.

Another important challenge in this research area is the development of large public image archives with images as representative of the world population as possible [33]. The existing image archives mainly contain skin lesions from light-skinned people. The images in the ISIC database, for example, come mainly from the United States, Europe, and Australia. To achieve an accurate classification for dark-skinned people as well, the CNN must learn to abstract from the skin color. However, this can only occur if it observes enough pictures of dark-skinned people during the training.

An improvement in classification quality could be achieved by adding clinical data (eg, age, gender, race, skin type, and anatomic location) as inputs for the classifiers. This additional information is advantageous for the decision making of dermatologists, as Haenssle et al [3] show. Future work should take these aspects into account.

### Conclusions

Unfortunately, it is difficult and in many times impossible to compare the performance of published classification results since many authors use nonpublic datasets for training and/or testing. Future publications should use publicly available benchmarks and fully disclose methods used for training to allow comparability.

## Abbreviations

AUC: area under the curve
CNN: convolutional neural network
FN: false negative
FP: false positive
ISBI: International Symposium on Biomedical Imaging
ISIC: International Skin Imaging Collaboration
ROC: receiver operating characteristic
TN: true negative
TP: true positive
